# Pleomorphic adenoma of hard palate: a case report

**DOI:** 10.11604/pamj.2021.38.146.26508

**Published:** 2021-02-09

**Authors:** Zemmouri Yousra, Chbicheb Saliha

**Affiliations:** 1 Département d´Odontologie Chirurgicale, Faculté de Médecine Dentaire de Rabat, Université Mohammed V, Rabat, Maroc

**Keywords:** Benign mixed tumour, hard palate, pleomorphic adenoma, salivary gland, case report

## Abstract

Pleomorphic adenoma is a benign mixed tumor, which is composed of myoepithelial and epithelial cells. A fibrous capsule separates these cells from the surrounding tissues. Pleomorphic adenoma is the most common salivary gland tumour accounting for 40-70% of all major and minor salivary gland tumours. It is also the commonest minor salivary gland benign tumours accounting for 70% of all tumours. Hard palate is the commonest site followed by upper lip, buccal mucosa, tongue, floor of mouth, retromolar trigone. This case report discusses a case of pleomorphic adenoma of hard palate in an old man after complete excision of the tumour, which was confirmed by a biopsy specimen.

## Introduction

Pleomorphic adenoma (PA) is the most common neoplasm of the large salivary glands and affects mostly the parotid gland, less frequently the accessory salivary glands. It derives its name from the architectural pleomorphism seen by light microscopy [1]. Tumors arising from the minor salivary glands are uncommon clinical entities, accounting for 10-25% of all salivary glands’ tumors. The palate is the most common site amongst the minor salivary glands for pleomorphic adenoma to occur, but they can also occur in the upper lip, cheek, floor of mouth, larynx and trachea [2]. The aim of this paper is to describe a case of pleomorphic adenoma of minor salivary gland in the palate of an old man patient who was treated with surgical excision of tumour showing no evidence of recurrence one-year post-operative follow-up.

## Patient and observation

A 38-year-old male patient reported to the department of Oral Surgery of the Consultation Center of Dental Treatment (CCDT) in Rabat, Morocco. The patient´s chief concern was swelling in his upper left back tooth region. History revealed the swelling was painless and gradually grew over one year to its present size. On general examination, all the vital signs were within the normal range with no history of diabetes or hypertension. Past dental history revealed extractions of 23, 24, 26 and 27 two years prior to presentation.

**Figure 1:**
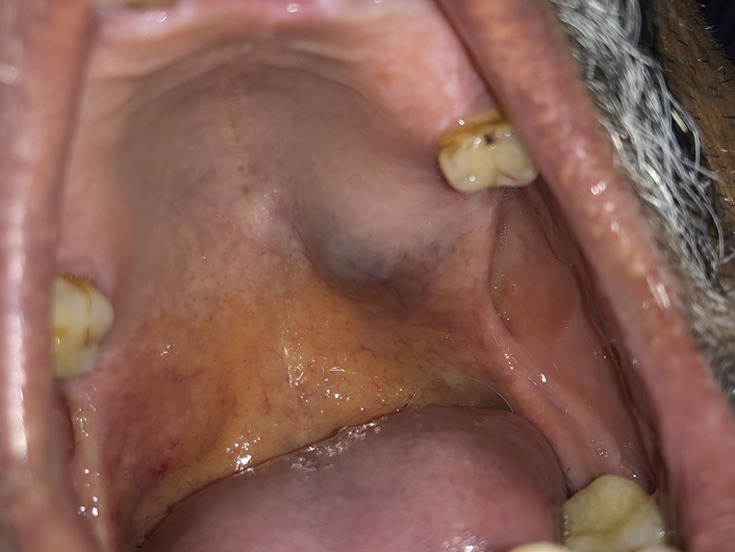
pre-operatory view of the lesion showing the left hard palate swelling

On intraoral examination, we noted a single, ovoid-shaped swelling measuring 2cm x 2cm in the left posterolateral surface of the hard palate. Medially, it extended from the midline of the hard palate and distal aspect of the region of 27 laterally (Figure 1). The overlying mucosa appeared healthy and smooth with a bluish appearance. On palpation, the swelling was unilocular, nontender, nonpulsatile, firm and immovable with well-defined margins. The mucosa over the lesion was stretched and nonpinchable.

**Figure 2:**
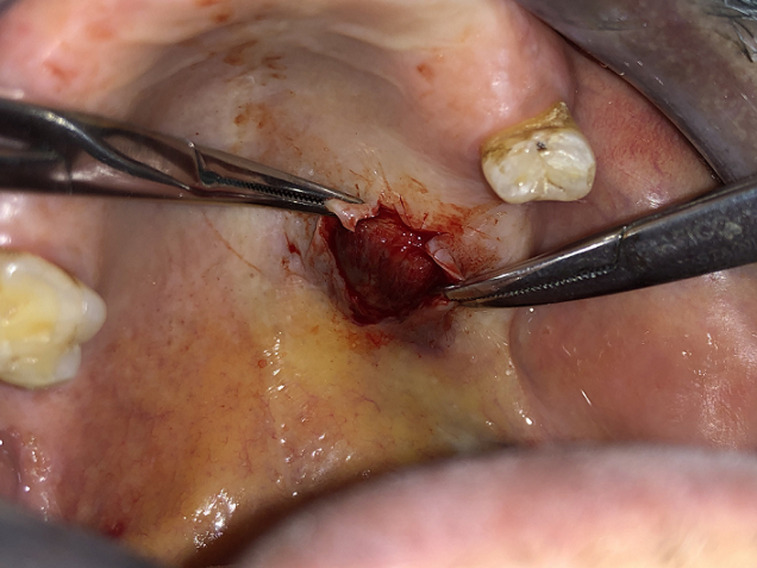
intraoperative clinical picture showing mass being separated from underlying bone

**Figure 3:**
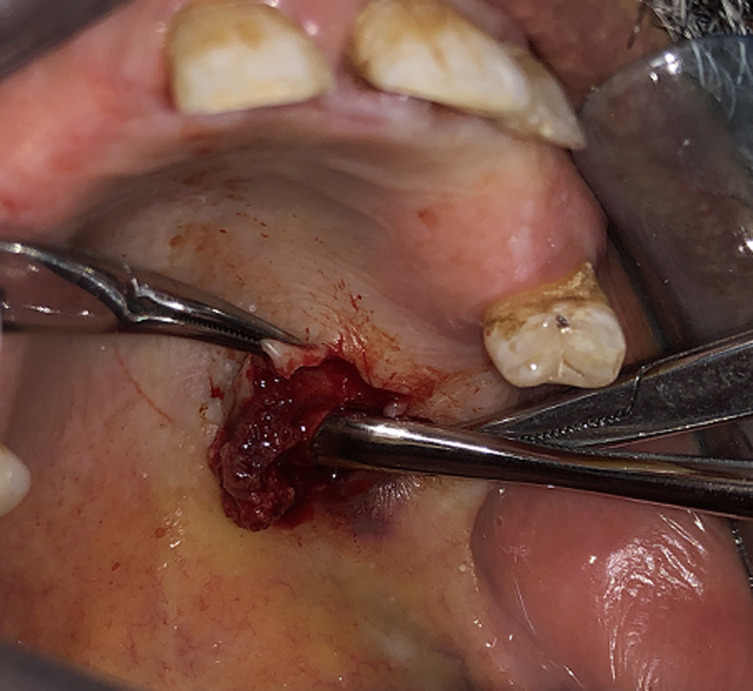
excised specimen of pleomorphic adenoma of hard palate

**Figure 4:**
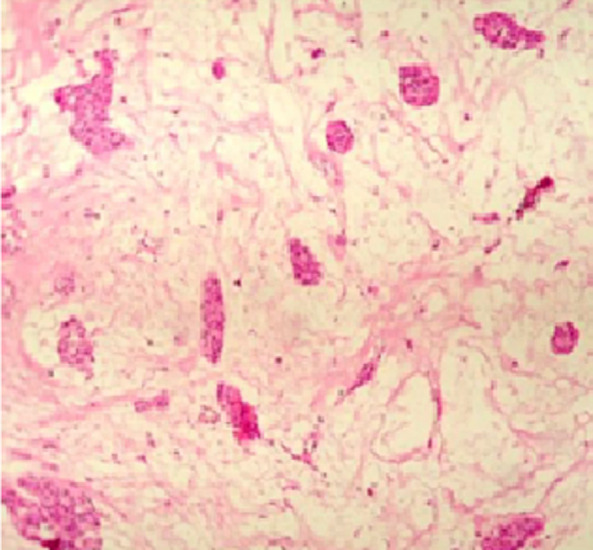
histology of the tumor showing the ductal epithelial and myoepithelial elements with chondro-myxoid stroma (H&E, 10X)

On radiological investigation, there was solitary heterogenous swelling in the right hard palate without calcification and bony erosion. With all this findings, provisional diagnosis of pleomorphic adenoma of hard palate was made and planned for surgical excision. Surgical excision of the mass was done in total along with the overlying mucosa and taking margin from surrounding mucosa (Figure 2). The lesion was in the form of an ovoid well demarcated, partially encapsulated, red-white partly myxoid, partly rubbery mass, measuring 1 x 1 x 1.5cm, with solid cut surface (Figure 3). The result of the histopathological examination was compliant with the specimen taken before the surgery and confirmed the diagnosis of pleomorphic adenoma (Figure 4).

## Discussion

The prevalence of tumors in small salivary glands accounts for 20-40%. The smaller the salivary gland that is affected, more likely it shows malignant behavior. Most commonly affected age group of patients is the ones between the fourth to sixth decades, predominantly the females. Mainly it occurs on the hard and soft palate because majority of the minor salivary glands are located in this area. Pleomorphic adenoma usually presents as a progressive slow growing swelling which is asymptomatic and firm in consistency [3]. Pleomorphic adenoma has a different embryological origin. It arises from both epithelial and mesenchymal origin. They arise from intercalated and myoepithelial cells. The mass is well demarcated from surroundings by fibrous capsule. Formation of the capsule is a result of fibrosis of the surrounding salivary parenchyma which is composed of the tumor and is referred to as false capsule. The pleomorphic adenoma is typically a well circumscribed, encapsulated tumor. The capsule may be incomplete which is more common in minor salivary gland tumours [4].

Diagnosis is based on the history, physical examination, radiological investigation and histopathological examination report. On examination, the differential diagnosis includes palatal abscess, odontogenic or non-odontogenic cyst, soft tissue tumor such as neurofibroma, fibroma, neurilemmoma [3]. Palatal abscess can be excluded by examining because it arises from non-vital tooth in the surrounding defect. The odontogenic and nonodontogenic cysts can be excluded during exploration of mass as it does not reveal its cystic consistency. Myoepithelioma have spindle shape cells and is a benign epithelial salivary gland tumor [5].

Radiographically, a computerized tomography (CT) scan would be ideal to determine extent of lesion, bony erosion and invasion, whereas magnetic resonance imaging (MRI) would help delineate soft tissue spread [6]. Histologically, it reveals epithelial and myoepithelial elements arranged in different patterns in mucopolysaccharide stroma. False capsule may be seen which forms as a result of fibrosis of surrounding salivary parenchyma that got compressed due to tumour [6]. The treatment of choice for pleomorphic adenoma should be wide local excision with the removal of periosteum or bone if they are involved. Simple enucleation of this tumor may lead to high recurrence rate and should be avoided [7].

Palatal reconstruction is considered in cases of large palatal defects arising after surgical excision in very aggressive tumors. In the present case, the patient did not require any reconstruction of the palate as the bony invasion was minimal which leads to regeneration of the palatal mucosa without any fistula formation [8]. Recurrence rates of these tumours are not seen, if adequate surgical excision has been performed [9]. Recurrences can occur with enucleation procedures, where the chances of leaving pseudo pod like microscopic extensions is a possibility due to the absence of a true capsule in these cases. A recurrence rate of 6% has been noted by Spiro in his evaluation of 1342 patients with benign minor salivary gland neoplasms [10].

## Conclusion

Pleomorphic adenoma of minor salivary gland is relatively rare, then as early as possible, a diagnosis should be established. Complete excision of the lesion is a definitive treatment protocol for these cases. However, one should try and prevent breach in the continuity of lesion and remove the entire lesion in toto, to minimize recurrence and transformation into malignancy.
